# Development and Application of a Quantitative PCR Assay to Assess Genotype Dynamics and Anatoxin Content in *Microcoleus autumnalis*-Dominated Mats

**DOI:** 10.3390/toxins10110431

**Published:** 2018-10-26

**Authors:** Laura T. Kelly, Susanna A. Wood, Tara G. McAllister, Ken G. Ryan

**Affiliations:** 1 School of Biological Sciences, Victoria University of Wellington, P.O. Box 600, Wellington 6140, New Zealand; ken.ryan@vuw.ac.nz; 2 Cawthron Institute, Private Bag 2, Nelson 7042, New Zealand; susie.wood@cawthron.org.nz; 3 Te Pūnaha Matatini, University of Auckland, Auckland 1142, New Zealand; tara.mcallister0@gmail.com

**Keywords:** *anaC* gene, benthic, rivers, cyanobacteria

## Abstract

*Microcoleus* is a filamentous cyanobacteria genus with a global distribution. Some species form thick, cohesive mats over large areas of the benthos in rivers and lakes. In New Zealand *Microcoleus autumnalis* is an anatoxin producer and benthic proliferations are occurring in an increasing number of rivers nationwide. Anatoxin content in *M. autumnalis*-dominated mats varies spatially and temporally, making understanding and managing proliferations difficult. In this study a *M. autumnalis*-specific TaqMan probe quantitative PCR (qPCR) assay targeting the *anaC* gene was developed. The assay was assessed against 26 non-*M. autumnalis* species. The assay had a detection range over seven orders of magnitude, with a limit of detection of 5.14 × 10^−8^ ng μL^−1^. The *anaC* assay and a cyanobacterial specific 16S rRNA qPCR were then used to determine toxic genotype proportions in 122 environmental samples collected from 19 sites on 10 rivers in New Zealand. Anatoxin contents of the samples were determined using LC-MS/MS and anatoxin quota per toxic cell calculated. The percentage of toxic cells ranged from 0 to 30.3%, with significant (*p* < 0.05) differences among rivers. The anatoxin content in mats had a significant relationship with the percentage of toxic cells (*R*^2^ = 0.38, *p* < 0.001), indicating that changes in anatoxin content in *M. autumnalis*-dominated mats are primarily related to the dominance of toxic strains. When applied to more extensive samples sets the assay will enable new insights into how biotic and abiotic parameters influence genotype composition, and if applied to RNA assist in understanding anatoxin production.

## 1. Introduction

Benthic cyanobacterial proliferations in freshwater ecosystems, in particular *Microcoleus* spp. and closely related taxa, pose a significant risk to ecosystem, animal, and human health [1,2,3,4]. Several *Microcoleus* and closely related species produce the potent neurotoxins anatoxin-a (ATX) and homoanatoxin-a (HTX), and their dihydro-variants; dihydroanatoxin-a (dhATX) and dihydrohomoanatoxin-a (dhHTX; [1,5], hereafter collectively referred to as anatoxins). While the toxic effects of consumption of anatoxins are known for some vertebrates, e.g., dogs, their impact on aquatic ecosystems remains unclear [6]. 

A significant factor impeding advancements in knowledge on the ecological function of anatoxins and the ability to predict its concentrations in *Microcoleus*-dominated samples is the coexistence of multiple strains with varying anatoxin quota (concentration of anatoxin per cell; [1,7]). Some strains do not carry the gene cluster (*anaA–anaK*; [8]) responsible for anatoxin synthesis, while others carry the gene cluster but produce different quantities of each anatoxin variant [1,7,9]. The co-existence of toxic and non-toxic strains suggests that the benefits of producing anatoxins do not always outweigh the costs associated with anatoxin production. Improving knowledge of the conditions that favour toxic *Microcoleus* strains has the potential to help predict when proliferations are more likely to be toxic. Research in this area has been limited by a lack of tools to quantify toxic and non-toxic *Microcoleus* genotypes in environmental samples. Morphologically, the strains appear identical, and while it is possible to test for the presence of the *ana*-gene cluster, to date the proportion of toxic and non-toxic genotypes in environmental samples has not been assessed [10]. 

In New Zealand, *Microcoleus autumnalis* (formerly *Phormidium autumnale*) is an anatoxin producer and proliferations have been reported in an increasing number of rivers nationwide [11]. Anatoxin content in *M. autumnalis*-dominated mats exhibit remarkable spatial and temporal variability [4,12,13,14]. There are several explanations for this: that the composition of non-*Microcoleus* mat components (e.g., sediment, microbial community) varies over time and between sites [15,16]; physicochemical factors within the mats or the overlying water result in up- or down-regulation of anatoxin production [17]; and toxic and non-toxic genotypes co-occur in the mats and the relative abundance of these genotypes influences the overall anatoxin content in the mats [7,13]. Wood and Puddick [13] found that anatoxin concentrations closely followed the abundance of copies of anatoxin genes in environmental samples, however, their study did not examine whether the proportion of toxic and non-toxic strains varied. The need for a quantitative method to determine the relative abundance of toxic to non-toxic *M. autumnalis* has been highlighted by numerous studies [6,11,13,18]. The present study aimed to develop a *M. autumnalis*-specific quantitative PCR (qPCR) assay to quantify *anaC* gene copies in environmental samples. This assay was then applied, in combination with a cyanobacterial specific 16S rRNA assay [19], to environmental samples enabling the proportion of toxic gene copies to total cyanobacterial gene copies to be determined. This data will enhance understanding of how anatoxin quota and the proportion of toxic genotypes varies within and between rivers, with a view to informing future management of proliferation events.

## 2. Results

### 2.1. Quantitative PCR

Species-specific primers and a probe targeting a highly conserved 100 bp region of the *M. autumnalis anaC* gene were designed. The sequences for the primers and probe were: Phor-AnaC-F5 5′-ACTAACCGAATCACTTCCACTT-3′, reverse primer: Phor-AnaC-R5 5′-CTCACCCACCTCACCTTTAG-3′, probe: Phor-AnaC-P5 5′-TTCAGTATTAGCGCAGGCTTTGCC-3′. The probe had the fluorescent reporter dye FAM-6-carbyfluoroscein at the 5′ end and was labelled with the non-fluorescent Black Hole Quencher^®^-1 (IDT, Coralville, IA, USA). Primer and probe sequences were checked for potential cross-reactivity in GenBank using the BLAST online software (http://blast.ncbi.nlm.gov/Blast.cgi) and no cross-reactivity was detected. *In vitro* tests of specificity did not result in amplification of any non-target species, including a strain of *Cuspidothrix issatchenkoi* (which is the only other known anatoxin producer in New Zealand), three *Aphanizomenon*, two *Aphanocapsa*, two *Raphidiopsis raciborskii*, eight *Dolichospermum*, two *Leptolyngbya*, two *Microcystis*, one *Nostoc*, two *Oscillatoria* (both anatoxin producers), one *Planktothrix,* and two *Scytonema* (Table A1). The assay had a linear range of detection over seven orders of magnitude, with the limit of detection reached at 5.14 × 10^−8^ ng μL^–1^ DNA.

### 2.2. Environmental Samples

**Figure 1:**
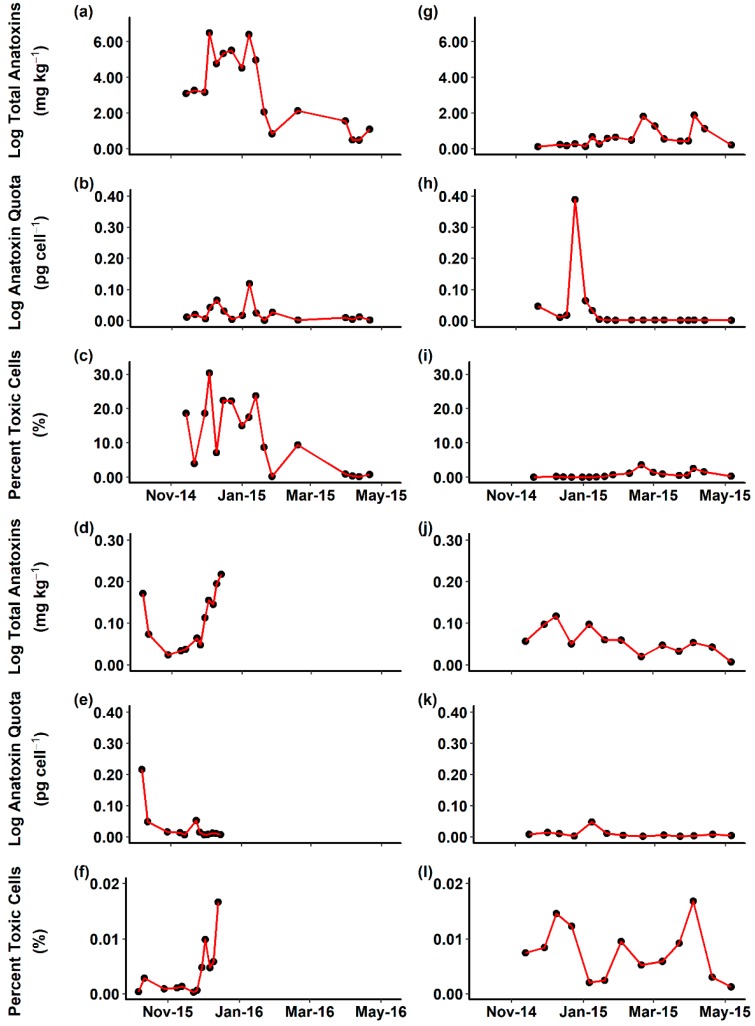
Total anatoxins, anatoxin quota and percent toxic cells for (**a**–**c**) Ashley; (**d**–**f**) Maitai; (**g**–**i**) Opihi; (**j**–**l**) Temuka rivers between October 2014 and December 2015. All points reflect a pooled 10-mat sample from each river on that sampling date. Note different y-axis scales between (**a**–**c**)/(**g**–**i**) and (**d**–**f**)/(**j**–**l**).

Samples from 19 sites in 10 rivers (n = 122) were analysed for anatoxin content using LC-MS/MS, and the quantity of *M. autumnalis anaC* and cyanobacterial specific 16S rRNA copies determined using qPCR. Anatoxin quota was determined by dividing weight normalised total anatoxin content by *anaC* copy numbers and the percentage of toxic cells in the total cyanobacterial community was calculated. There were no consistent temporal trends in anatoxin content, anatoxin quota or the percentage of toxic cells among the Ashley, Opihi, Maitai, and Temuka rivers (Figure 1). The total anatoxin content and percentage of toxic cells were higher in the Ashley and Opihi rivers than in the Maitai and Temuka rivers. The Ashley River had the greatest variability in both total anatoxin content and percentage of toxic cells, ranging from 0.6 to 662.5 mg kg^−1^ and 0.1 to 30.3% respectively. In this location, there was a trend from higher and more variable anatoxin content and percentage of toxic cells early in the season, to lower and less variable anatoxin content and percentage of toxic cells in March–April 2015. In contrast, the Maitai River had a relatively high anatoxin content and a lower percentage of toxic cells in October 2015. Both the Opihi and Temuka rivers exhibited variable anatoxin content and percentage of toxic cells with no apparent temporal trends. Anatoxin quota were variable for all rivers (Figure 1), however with the exception of a single occasion on the Opihi River, the anatoxin quota varied less than 2-fold.

**Figure 2:**
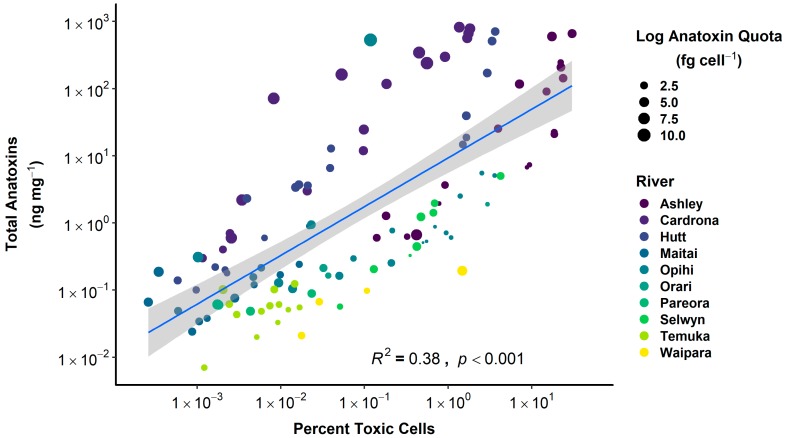
Relationship between percentage of toxic cells (determined using quantitative PCR) and total anatoxin content of samples. The blue line is a linear regression (±SE, grey shading). Colours represent rivers and the size of dots represent anatoxin quota (fg cell^−1^). The limit of detection/limit of quantitation of total anatoxins was 0.002/0.006 ng mg^−1^, respectively.

The total anatoxin content of samples was significantly correlated with the percentage of toxic cells (*R*^2^ = 0.38, *p* < 0.001, Figure 2). Samples from the Ashley, Cardrona, and Hutt rivers generally had higher anatoxin quota. Samples with a higher than expected anatoxin content relative to the percentage of toxic cells also had a higher anatoxin quota, while those with a lower than expected anatoxin content also had lower anatoxin quota. The proportion of each anatoxin congener (ATX, HTX, dhATX, and dhHTX) varied among all samples measured with the dihydro-congeners usually the most abundant (Figure S1).

**Figure 3:**
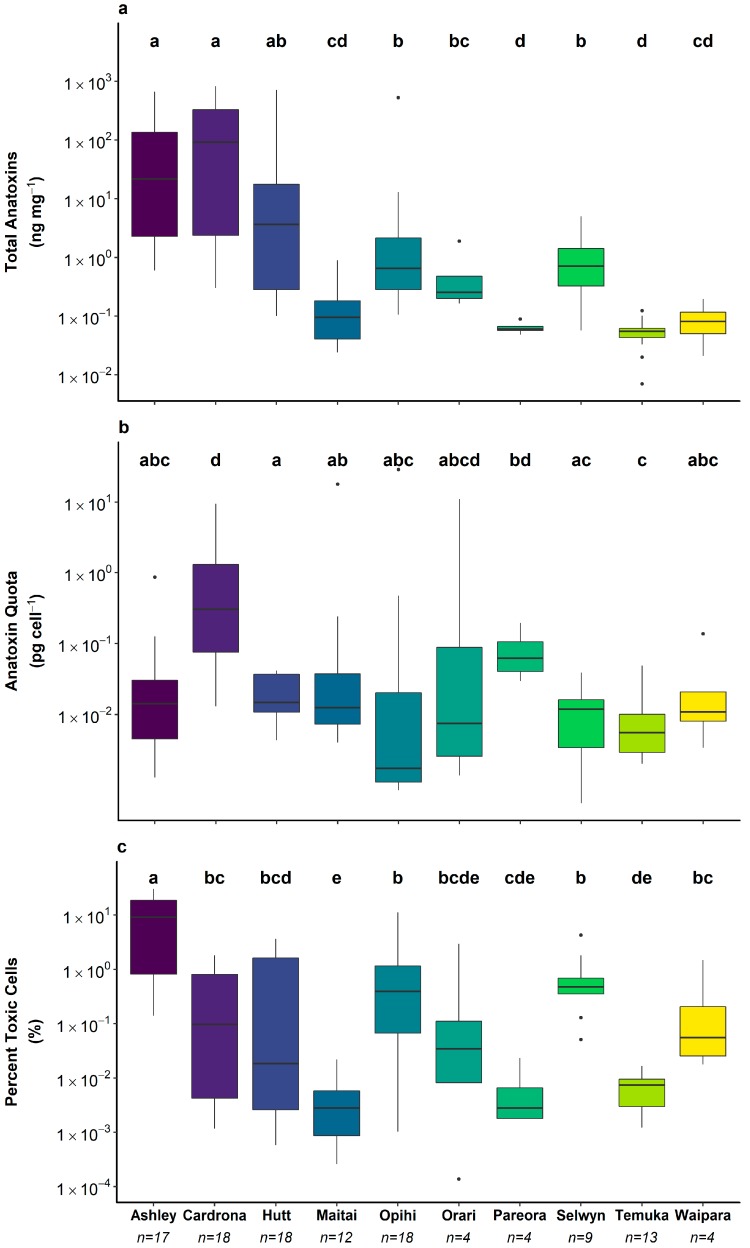
(**a**) total anatoxin concentration; (**b**) anatoxin quota and (**c**) percent toxic cells. Lines within the boxes are medians, the ends of boxes are quartiles and whiskers extend to the lowest or highest data point ≤ 1.5× interquartile range. Black dots are outliers. A Kruskal–Wallis test and pairwise Wilcoxon rank sum test with a Benjamini–Hochberg adjustment was used to identify rivers that were significantly different from one another (*p* < 0.05), denoted by the letter above the plot. Where timeseries data were available, these were pooled for this analysis. The limit of detection/limit of quantitation for anatoxins was 0.002 and 0.006 ng mg^−1^, respectively.

Anatoxin content of samples from the Ashley, Cardrona, and Hutt rivers spanned four orders of magnitude, while those of the other seven rivers generally had lower anatoxin contents and spanned only two orders of magnitude (Figure 3a). Statistically significant differences in anatoxin content were identified between rivers (pairwise Wilcoxon rank sum test, *p* < 0.05; Figure 3a). Anatoxin quota were highly variable within rivers, however, in contrast to the anatoxin content there was more overlap among rivers (Figure 3b). The median percentage of toxic cells was less than 1% in all rivers except the Ashley where it was 9.3% (Figure 3c). 

**Figure 4:**
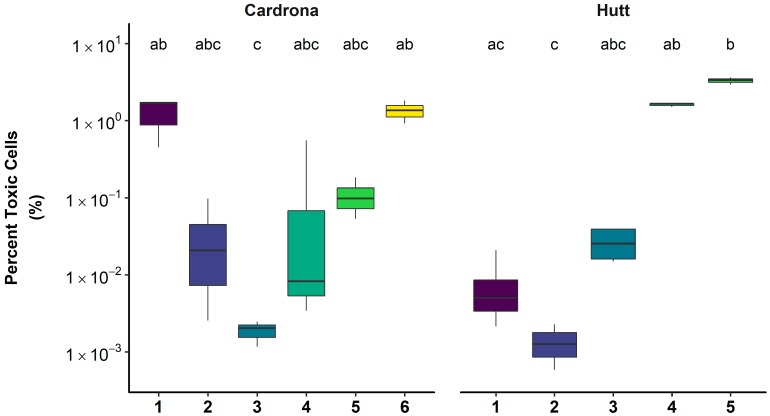
Percent toxic cells for sites along the Cardrona and Hutt rivers. See Figure 3 for interpretation of boxplots. Sites are in order from upstream to most downstream on each river (see Figure 5). A Kruskal–Wallis test and Dunn’s post-hoc test were used to identify significant differences (*p* < 0.05) among sites, denoted by the letter above the plot.

Within rivers, the percentage of toxic cells varied considerably, with the Ashley, Cardrona and Hutt rivers ranging from 0.1%–30.3%, 0.001%–1.8%, and 0.009%–3.6% toxic cells, respectively. Within the Hutt and Cardrona rivers, there were significant differences in the proportion of toxic cells among sites (Figure 4).

## 3. Discussion

*Microcoleus autumnalis* proliferations are increasing in prevalence and there is a growing need to understand more about anatoxin content variability and production [6,18]. The first aim of this study was to develop a quantitative PCR method to enumerate the copy numbers of *anaC* in *M. autumnalis*-dominated mat samples. When used in concert with LC-MS/MS analysis of anatoxins and a second qPCR enumerating the total 16S rRNA cyanobacterial copy numbers; this enables both anatoxin quota and the percentage of toxic cells to be determined. The *M. autumnalis*-qPCR assay was specific, sensitive and robust. Its specificity was verified using 24 non-*Microcoleus* cultures mostly isolated from New Zealand, including the only other known anatoxin producer in New Zealand (*Cuspidothrix issatchenkoi*), and no amplification was detected. An advantage of such a specific assay is that it may be applied in systems where multiple anatoxin producing species co-occur, enabling the quantitation of the *anaC* contained within *M. autumnalis.* The qPCR can easily transferrable to emerging technologies, including droplet digital PCR (ddPCR; [13]). An increasing number of countries are now identifying *M. autumnalis* as an anatoxin producer e.g., [20], therefore the assay may have widespread applicability to other regions of the world in the future.

The cyanobacterial 16S rRNA assay used in this study detects other non-*M.autumnalis* cyanobacteria in mat samples, thus the proportion of toxic cells determined in this study represents the proportion relative to the entire cyanobacterial community within the mat. Previous studies have shown that the abundance of other cyanobacteria in *M. autumnalis*-dominated mats is very low [16,21]. Therefore detections of other cyanobacteria are unlikely to have a marked impact on the relationships identified. In addition, the 16S rRNA primers were checked in silico using the BLAST online software (http://blast.ncbi.nlm.gov/Blast.cgi) to identify possible detections of diatoms. No cross-reactivity of the primers to the commonly occurring diatoms in *M. autumnalis*-dominated mats was detected, and cross-reactivity was detected with a single freshwater diatom; *Rhopalodia gibba*. Despite a lack of cross-reactivity, ideally, a *M. autumnalis*-specific reference qPCR would be used instead of a general cyanobacterial assay. Attempts made to develop such an assay during this study were unsuccessful as we were unable to amplify the genes of interest in *M. autumnalis* (*rpoC* and *cpcB*) to enable sequencing and primer design. Future work should include whole genome sequencing, which will yield sequences for assay development.

There was no relationship between the anatoxin quota and percentage of toxic cells in the Ashley, Opihi, Maitai, or Temuka rivers in multiple samples at different times, suggesting that anatoxin quota is not closely related to the percentage of toxic cells in *M. autumnalis*-dominated mats. This contrasts with *Raphidiopsis raciborskii*, where the cylindrospermopsin quota was highly correlated with the relative proportion of toxic gene copies [22]. Individual strains of *M. autumnalis* isolated from New Zealand rivers exhibit up to 100-fold differences in their total anatoxin content, in addition to producing different proportions of the four anatoxin-variants [7]. Environmental samples exhibited variation in the proportion of different anatoxin congeners produced, and consistent with previous work, the dihydro-congeners were dominant in most samples [4,7,13]. It is probable that the decoupling of anatoxin content and the anatoxin quota observed in this study reflects shifts in the dominance of different toxic strains within mats. The anatoxin content of *M. autumnalis*-dominated mats was highly variable within and between rivers, and over time, which is consistent with observations from previous studies [4,12,14]. Both anatoxin concentrations and percentage of toxic cells varied over time in the Ashley and Maitai rivers. While this is consistent with studies on other rivers in New Zealand [14], datasets spanning multiple years are required to confirm that this trend is consistent over multiple proliferation seasons. Identification of temporal trends in anatoxin content, anatoxin quota and the percentage of toxic cells in *M. autumnalis*-dominated mats is complicated by significant spatial heterogeneity. Often, anatoxin concentrations vary within a very small spatial area (i.e., 5–10 cm), and are significantly different over short time periods [13]. Sampling of *M. autumnalis*-dominated mats is destructive, and the presence of spatial heterogeneity may result in observations reflecting changes in spatial rather than temporal differences. The use of an experimental system where the inoculum source is controlled and known would help mitigate this issue and identify whether there are temporal or successional trends in the proportion of toxic cells in mats. 

The relationship between the total anatoxin content and percentage of toxic genotypes in the present study was significant. A relationship between the total abundance of toxic cells and anatoxin content in *M. autumnalis*-dominated mats has previously been identified [13], however, our study demonstrates that the proportion of toxic cells is also important. Similar correlations in microcystin concentrations and the proportion of toxic cells to the total cyanobacterial community in environmental samples have been observed in *Microcystis* and *Planktothrix*, though there the relationship was much stronger [23]. In *M. autumnalis*, anatoxin quota differ among toxic strains [7], thus some variation in the relationship between anatoxin content and percentage of toxic cells is to be expected. The changes in anatoxin content observed in this study may therefore reflect changes in the dominance of toxic strains with differing anatoxin producing ability. This is supported by the anatoxin quota data, which shows that samples with higher than expected anatoxin content relative to the proportion of toxic cells also had higher anatoxin quota; indicating that the dominant toxic strains in these samples produce more anatoxin than average. 

The percentage of toxic cells ranged from 0.1 to 30.3% in the Ashley River and from <0.01 to 3.6% across the other rivers. These results broadly align with similar studies in other genera including *Microcystis*; toxic *Microcystis* made up 12 to 100% of the total cells in Lake Ronkonkoma (New York) and 0.01 to 6% in other lakes [23], while toxic *Microcystis* comprised 0.01 to 27% of the total *Microcystis* population in San Francisco Estuary [24]. Similarly, while toxic cells of *Planktothrix* comprised between 75 and 100% of the *Planktothrix* community in the Masurian lakes (Poland), when compared to the total cyanobacterial community, toxic cells made up between 0.01 and 5.89% of the community [25]. Very little is understood about the dynamics of toxic and non-toxic strains of *M. autumnalis* in environmental samples because of the lack of tools to distinguish their contribution to the population. It is intriguing that the proportion of toxic cells in the *M. autumnalis*-dominated mats we tested never reached the high levels seen in some populations of *Microcystis* spp. and *Planktothrix* spp. This may be the result of taking pooled samples from multiple cobbles; *M. autumnalis*-dominated mats are known to exhibit high spatial variability in toxin content [4,13] and this could also be reflected in the proportion of toxic cells. There remains a need for future work to investigate the dynamics of toxic and non-toxic strains and how their proportions change in relation to mat development and environmental conditions. The large variability in the proportion of toxic cells in the Hutt and Cardrona rivers is reflected in considerable differences between samples collected from different sites on the same day. It is possible that the variation observed between rivers and sites in this study was the result of physicochemical parameters. Other genera of cyanobacteria including *Microcystis* and *R. raciborksii* exhibit variation in the relative abundance of toxic genotypes or toxin production under different nutrient levels [22,23,26]. Previous culture-based studies in *M. autumnalis* suggest that anatoxin quota are lowest under low nitrogen and phosphorus conditions [27] and that toxic strains may be favoured at temperatures greater than 13.4 °C [12]. The lack of available molecular methods has prevented the relationships between the proportion of toxic cells and physicochemical variables in environmental *M. autumnalis*-dominated mats from being determined until now. Future work should examine physicochemical variables across a range of rivers and over time to allow these relationships to be elucidated.

## 4. Conclusions

The qPCR assay developed in the present study enables the quantification of *M. autumnalis anaC* copy numbers in environmental samples. LC-MS/MS was used to determine anatoxin content and a second qPCR used to determine the total cyanobacterial 16S rRNA copy numbers in environmental samples. These data enabled anatoxin quota and the abundance of toxic/non-toxic strains to be determined in *M. autumnalis*-dominated mats. The results indicate that changes in anatoxin content in these mats are primarily related to the dominance of toxic strains. 

The *M. autumnalis anaC* qPCR assay now provides an opportunity to investigate the dynamics of toxic/non-toxic strain dominance in environmental samples and if used on RNA (via complementary DNA) could enable insights into anatoxin regulation in this species. Future studies should use fine-scale, standardised sampling across a range of physicochemical gradients to identify factors that influence the relative abundance of toxic and non-toxic strains and anatoxin production. 

## 5. Materials and Methods

### 5.1. Samples for Quantitative PCR Assay Development and Validation

*Microcoleus autumnalis* strains (n = 26) isolated from New Zealand rivers were sourced from the Cawthron Institute Culture Collection of Micro-algae (CICCM, http://cultures.cawthron.org.nz/; Table 1). Cultures were non-axenic. An additional 24 non-axenic cyanobacterial strains including non-*Microcoleus* anatoxin producers were sourced from CICCM (Table A1) for verification of assay specificity. Strains tested included: three *Aphanizomenon*, two *Aphanocapsa*, one *Cuspidothrix*, two *R. raciborskii*, eight *Dolichospermum*, two *Leptolyngbya*, two *Microcystis*, one *Nostoc*, one *Planktothrix,* and two *Scytonema*. Sub-samples (ca. 200 mg wet weight) of cultures were harvested into a 1.7 mL microcentrifuge tube and centrifuged (12,000× *g*, 1 min). The supernatant was discarded and pellets frozen (−20 °C) until extraction. DNA was extracted using a Purelink^®^ Genomic DNA extraction kit (Thermo Fisher Scientific, Waltham, MA, USA) following the manufacturer’s instructions. 

### 5.2. Primer Design and Optimisation for Quantitative PCR Analyses

Potential primer sites for detection of *M. autumnalis*-specific *anaC* gene were identified using a multiple sequence alignment (ClustalW; Thompson et al., 1994 [29]) of a ca. 300 base pair (bp) region within the *anaC* gene from six *M. autumnalis* and five *Oscillatoria* sp. sequences obtained from GenBank (www.ncbi.nlm.nih.gov) and Wood, Puddick, Fleming and Heussner [28]; Table A2. Species-specific primers and a probe targeting a 100 bp region of the *anaC* gene in *M. autumnalis* were designed. Primer and probe sequences were checked in silico for potential cross-reactivity in GenBank using the BLAST online software (http://blast.ncbi.nlm.gov/Blast.cgi) and then synthesized by IDT DNA (Coralville, IA, USA). Primers and probe concentrations were optimized for use with the Rotor-Gene-Q real-time rotary analyser (Qiagen, Venlo, The Netherlands) using *M. autumnalis* genomic DNA isolated from CICCM cultures CAWBG556 and CAWBG37. The optimized assay consisted of a 10 µL reaction containing; 5.6 µL KAPA Probe Fast QPCR Kit Master Mix (2×), 0.5 µL of primers (10 µM), 0.3 µL TaqMan probe, 2.1 μL Bovine serum albumin (BSA; Sigma, St. Louis, MO, USA) and 1 µL of template DNA. The optimised cycling profile was 95 °C for 3 min, followed by 40 cycles at 95 °C for 3 s and 60 °C for 20 s. Five-point standard curves ranging from 1.85 × 10^2^ to 10^5^ gene copies per μL and no template controls were analysed in triplicate on each qPCR run. The standard curve was constructed using purified (AxyPrep PCR Clean-up Kit, Axygen Biosciences, Union City, CA, USA) PCR product using the *M. autumnalis*-specific *anaC* primers described above. Duplicate reactions of PCR product (5.14 × 10^−2^ to 5.14 × 10^−9^ ng μL^−1^) were used to determine the limits of detection (LOD). The number of copies in the PCR product for the standard curves were determined using the Equation (1):

(1) $$anaC\;copies\;{\mu L}^{-1}=\frac{\left(A\times 6.022\times 10^{23}\right)}{\left(B\times 1\times 10^{9}\times 650\right)}$$

where A is the concentration of the PCR product and B is the length of the PCR product. The standard curves were linear (*R*^2^ > 0.99) and the qPCR efficiency ranged from 0.94 to 1.08. 

### 5.3. Environmental Sample Collection

**Figure 5:**
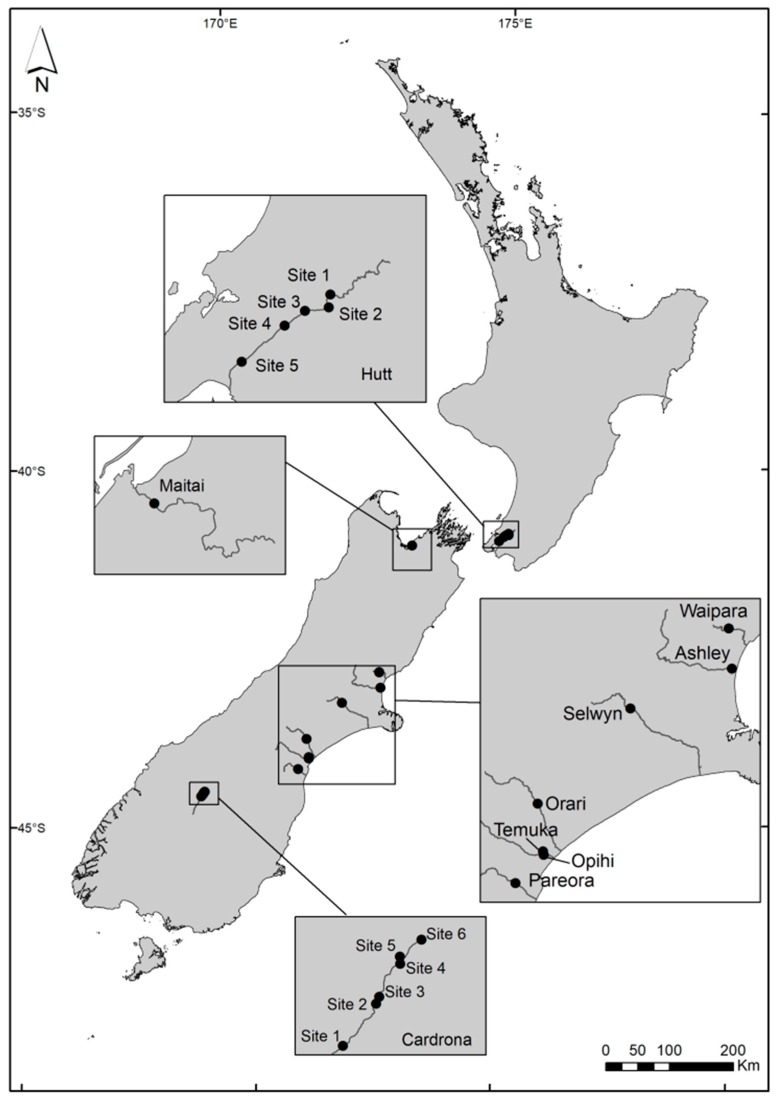
Sampling locations on 10 rivers in New Zealand. Sites 1–5 and 1–6 on the Hutt and Cardrona rivers, respectively, were collected from upstream to downstream on a single sampling occasion. Samples collected from the other eight rivers were collected over time from the same location.

Environmental samples from previous studies [18,28] were used to explore the variability in anatoxin quota and relative abundance of genotypes among 10 New Zealand rivers (n = 122, Figure 5). The samples were collected from 10 rivers throughout New Zealand. Samples from the eight rivers in Canterbury and the Maitai River (Nelson) consisted of pooled subsamples (~2 cm^2^) of mats from ten different cobbles at each site into a single collection tube. Samples from the Hutt River and Cardrona River consisted of a mat sample from a single cobble harvested into a collection tube. Samples were stored in the dark and on ice and frozen (−20 °C) on return to the laboratory. Four rivers (Ashley, Opihi, Maitai and Temuka) were chosen to conduct a time-series analysis to identify changes in the proportion of toxic strains and anatoxin quota over time. A further two of the rivers (Hutt and Cardrona) were sampled at multiple sites along the river, enabling a within-river comparison of the proportion of toxic strains and anatoxin quota.

### 5.4. DNA Extraction and Test for Inhibition

Environmental samples were lyophilised and homogenised. A sub-sample of approximately 20 mg was weighed and extracted using a PowerSoil^®^ DNA Isolation Kit (Qiagen, Redwood, CA, USA) following the manufacturer’s directions. All extracted DNA samples were aliquoted into two sub-samples and stored frozen (−20 °C) until use. Samples were screened for the presence of inhibitors using an internal control inhibition assay [30]. The assay consisted of an 11 μL reaction containing 6.25 μL KAPA Probe Fast qPCR Kit Master Mix (2×), 0.5 μL of each forward and reverse primer targeting the rRNA internal transcribed spacer region 2 (ITS2) of *Oncorhynchus keta* salmon sperm (10 μM, Sketa F2 and Sketa R3, IDT, Coralville, IA, USA, Haugland, Siefring, Wymer, Brenner and Dufour [30]), 0.2 μL TaqMan probe (10 μM) labelled with FAM-6-carboxyfluorescein at the 5′ end and with the Black Hole Quencher^®^-1 (IDT, Coralville, IA, USA) at the 3′ end, 1.55 μL DNA/RNA free water (Thermo Fisher Scientific, Waltham, MA, USA), 1 μL salmon sperm DNA (Sigma, St. Louis, MO, USA) and 1 μL of template DNA. The cycling profile was: 95 °C for 3 min, followed by 40 cycles at 95 °C for 3 s and 60 °C for 20 s. All samples showed inhibition and were diluted 1:10 with DNA/RNA free water (Thermo Fisher Scientific) and reanalysed for inhibition as above. 

### 5.5. Quantitative PCR of 16S rRNA Reference Gene

This qPCR assay was used to determine the number of cyanobacterial 16S rRNA gene copies in each sample. Reactions consisted of 5 μL Kapa SYBR Green Super Mix, 0.4 μL of each forward and reverse primer (cyano-real 16sF/R; Al-Tebrineh, et al. [31]), 2 μL BSA (Sigma), 1.2 μL DNA/RNA free water (Thermo Fisher Scientific), and 1 μL template DNA. The qPCR cycling conditions were the same as described for the *anaC* qPCR assay. Each reaction was run in triplicate using the optimised assay described above. Five-point standard curves ranging from 6.18 × 10^3^ to 10^7^ copies per μL and no template controls were analysed in triplicate on each qPCR run. The standard curves were constructed using purified (Nucelospin PCR Cleanup Kit, Machery-Nagel, Düren, Germany) PCR product using cyanobacterial 16S rRNA primers: 27F1 5′-AGAGTTTGATCCTGGCTCAG-3′ and 809R 5′-GCTTCGGCACGGCTCGGGTCGATA-3′ [32]. The standard curve was linear (*R*^2^ > 0.99) and the qPCR efficiency ranged from 0.82 to 0.93.

### 5.6. Anatoxin Extraction and Analysis

*Microcoleus autumnalis*-dominated mat samples were lyophilised and homogenised using a sterile metal spatula. Anatoxins were extracted from a known mass according to McAllister, Wood, Atalah and Hawes [18]. Each anatoxin extract was analysed for ATX, HTX, dhATX and dhHTX by liquid chromatography-tandem mass spectroscopy (LC-MS/MS), as described in Wood et al., 2017 [28]. An external standard curve consisting of dilutions of a certified ATX reference material (National Research Council, Ottawa, ON, Canada; 0.5–20 ng mL^−1^ in 0.1% formic acid) was used to determine ATX concentrations. Anatoxin was used as a calibration reference and a relative response factor of 1 used to quantify HTX, dhATX and dhHTX. The analytical limit of detection (LoD) was 0.02 ng mL^−1^ and limit of quantitation (LoQ) 0.06 ng mL^−1^. Detection limits in the extracted samples were 0.002 ng mg^−1^ (LoD) and 0.006 ng mg^−1^ (LoQ). The LC-MS/MS results (ng mL^−1^) were divided by the weight of the lyophilised sample (mg) to convert the data to ng mg^−1^.

### 5.7. Data and Statistical Analysis

The number of copies of the *anaC* and cyanobacterial 16S rRNA genes per microlitre of DNA extract was calculated as described in Equation (1). The results were converted to copies mg^−1^ using Equation (2).

(2) $$copies\;{\mathrm{mg}}^{-1}=\frac{\left(copies\;{\mu L}^{-1}\times C\times D\times E\right)}{weight\;of\;starting\;material\;\left(\mathrm{mg}\right)}$$

where *C* is the volume of the qPCR reaction, *D* is the dilution factor and *E* is the elution volume. Anatoxin quota per toxic cell were calculated using Equation (3).

(3) $$anatoxin\;quota\;\left(\mathrm{pg}\;anaC^{-1}\right)=\frac{\left[ATX+HTX+dhATX+dhHTX\right]\;\mathrm{pg}\;{\mathrm{mg}}^{-1}}{\left(anaC\;copies\right)\;{\mathrm{mg}}^{-1}}$$

where [ATX, HTX, dhATX, and dhHTX] are the concentrations of the respective anatoxins congeners (in pg) per mg of lyophilized mat material. To determine the ratio of toxic to non-toxic strains in the samples, their relative percentage was calculated by assuming that there are on average 2.3 16S rRNA copies per cyanobacterial genome and using Equation (4) [33].

(4) $$percent\;toxic\;strains=\left(\frac{anaC\;copies}{16S\;\mathrm{rRNA}\;copies÷2.3}\right)\times 100$$

Samples from the 10 study rivers were compared to examine differences in the proportion of toxic strains and anatoxin quota between rivers. All analyses were conducted in R (Version 3.1.3). The relationship between total anatoxins and the percentage of toxic genotypes across all samples was explored using a linear regression model, with the response variables log-transformed. Mean total anatoxins, anatoxin quota and proportion of toxic strains were analysed using a Kruskal–Wallis test and pairwise Wilcoxon rank sum test with a Benjamini–Hochberg adjustment as the data was non-normally distributed and had heterogeneity of variances. A within-river comparison of the anatoxin quota and proportion of toxic strains was undertaken for five sites on the Hutt and six sites on the Cardrona rivers using a Kruskal–Wallis Test. Pairwise differences between sites were explored with Dunn’s post-hoc test.

## Figures and Tables

**Table 1:** Presence and absence of *anaC* and *anaF* genes and anatoxins in *Microcoleus autumnalis* strains used in this study. Anatoxins were measured using liquid chromatography-tandem mass spectroscopy (LC-MS/MS; Wood et al., 2017 [28]).

Strain	*AnaC*	*AnaF* *	Anatoxins ** (LC-MS/MS)
CAWBG24	−	nt	−
CAWBG26	−	nt	−
CAWBG32	+	nt	nt
CAWBG36	−	nt	nt
CAWBG37	−	nt	nt
CAWBG38	−	nt	−
CAWBG46	−	nt	−
CAWBG48	−	nt	−
CAWBG50	−	nt	−
CAWBG51	−	nt	−
CAWBG52	−	nt	−
CAWBG53	−	nt	−
CAWBG54	−	nt	−
CAWBG55	−	nt	−
CAWBG56	−	nt	nt
CAWBG57	−	nt	nt
CAWBG58	+	nt	nt
CAWBG71	−	nt	nt
CAWBG503	+	+	+
CAWBG507	−	−	−
CAWBG511	−	−	−
CAWBG512	−	−	−
CAWBG520	+	+	+
CAWBG521	+	+	+
CAWBG556	+	+	+
CAWBG557	+	+	+

* *anaF* detection by Wood et al., 2012 and 2017 [7,28]; ** Anatoxin detection from Wood et al. [4]; nt indicates samples were not tested for this attribute.

## Supplementary Materials

The following are available online at http://www.mdpi.com/2072-6651/10/11/431/s1, Figure S1: Proportions of each of the four anatoxin congeners anatoxin (ATX), homoanatoxin (HTX), dihydroanatoxin (dhATX) and dihydrohomoanatoxin (dhHTX) for each of the environmental samples. Sample ID are in order from the lowest anatoxin quota (sample ID 1) to highest anatoxin quota (sample ID 122).

## Appendix A

**Table A1:** Primer specificity was tested against DNA from a range of anatoxin producing and non-anatoxin producing cyanobacteria. The anatoxin producing *Cuspidothrix* is the only known non-*Microcoleus* anatoxin producer in New Zealand.

Species	Culture Identification	Anatoxin Producer	Reference
*Aphanocapsa* sp.	CAWBG585	−	
*Aphanocapsa* sp.	CAWBG615	−	
*Aphanizomenon* sp.	CAWBG588	−	
*Aphanizomenon* sp.	CAWBG594	−	
*Aphanizomenon* sp.	CAWBG01		
*Cuspidothrix issatchenkoi*	CAWBG02	+	[34]
*Dolichospermum circinale*	CAWBG571	−	
*Dolichospermum circinale*	CAWBG601	−	
*Dolichospermum lemmermanii*	CAWBG572	−	
*Dolichospermum lemmermanii*	CAWBG593	−	
*Dolichospermum planktonicum*	CAWBG589	−	
*Dolichospermum spiroides*	CAWBG558	−	
*Dolichospermum spiroides*	CAWBG583	−	
*Dolichospermum* sp.	CAWBG608	−	
*Leptolyngbya* sp.	CAWBG586	−	
*Leptolyngbya* sp.	CAWBG700	−	
*Microcystis* sp.	CAWBG580	−	
*Microcystis* sp.	CAWBG561	−	
*Nostoc* sp.	S. Wood submitted	−	
*Oscillatoria* sp.	PCC 6506	+	[35]
*Oscillatoria* sp.	PCC 9029	+	[36]
*Planktothrix* sp.	CAWBG616	−	
*Raphidiopsis raciborskii*	CAWBG598	−	
*Raphidiopsis raciborskii*	CAWBG614	−	
*Scytonema* sp.	CAWBG699	−	
*Scytonema* cf. *crispum*	CAWBG72	−	

**Table A2:** Sequences from *Microcoleus autumnalis* and *Oscillatoria* sp. were used to identify a region of the *anaC* gene that is conserved within *M. autumnalis* but not within *Oscillatoria*, to generate *M. autumnalis* specific primers.

Species	Culture Identification	Genbank Accession Number (16S rRNA Gene)	Reference
*Microcoleus autumnalis*	CAWBG 618	KX016036	[28]
*M. autumnalis*	CAWBG 619	KX016037	[28]
*M. autumnalis*	CAWBG 620	KX016038	[28]
*M. autumnalis*	CAWBG 621	KX016039	[28]
*M. autumnalis*	CAWBG 622	KX016040	[28]
*M. autumnalis*	CAWBG 623	KX016041	[28]
*Oscillatoria* sp.	PCC 10111	JF803654.1	[10]
*Oscillatoria* sp.	PCC 10601	JF803652.1	[10]
*Oscillatoria* sp.	PCC 6407	JF803648.1	[10]
*Oscillatoria* sp.	PCC 9240	JF803651.1	[10]
*Oscillatoria* sp.	PCC 9240-strain 193	JF803653.1	[10]
