# Supplementary material for: Development and Application of a Quantitative PCR Assay to Assess Genotype Dynamics and Anatoxin Content in Microcoleus autumnalis-Dominated Mats
Source: Toxins (Basel). 2018 Oct 26;10(11):431. doi: 10.3390/toxins10110431 (PMC6266952; doi:10.3390/toxins10110431)
Supplement: Supplementary file 1 [file toxins-10-00431-s001.pdf]

Supplementary Materials: Development and Application of a Quantitative PCR Assay to Assess Genotype Dynamics and Anatoxin Content in *Microcoleus autumnalis*-Dominated Mats

Laura T. Kelly, Susanna A. Wood, Tara G. McAllister and Ken G. Ryan

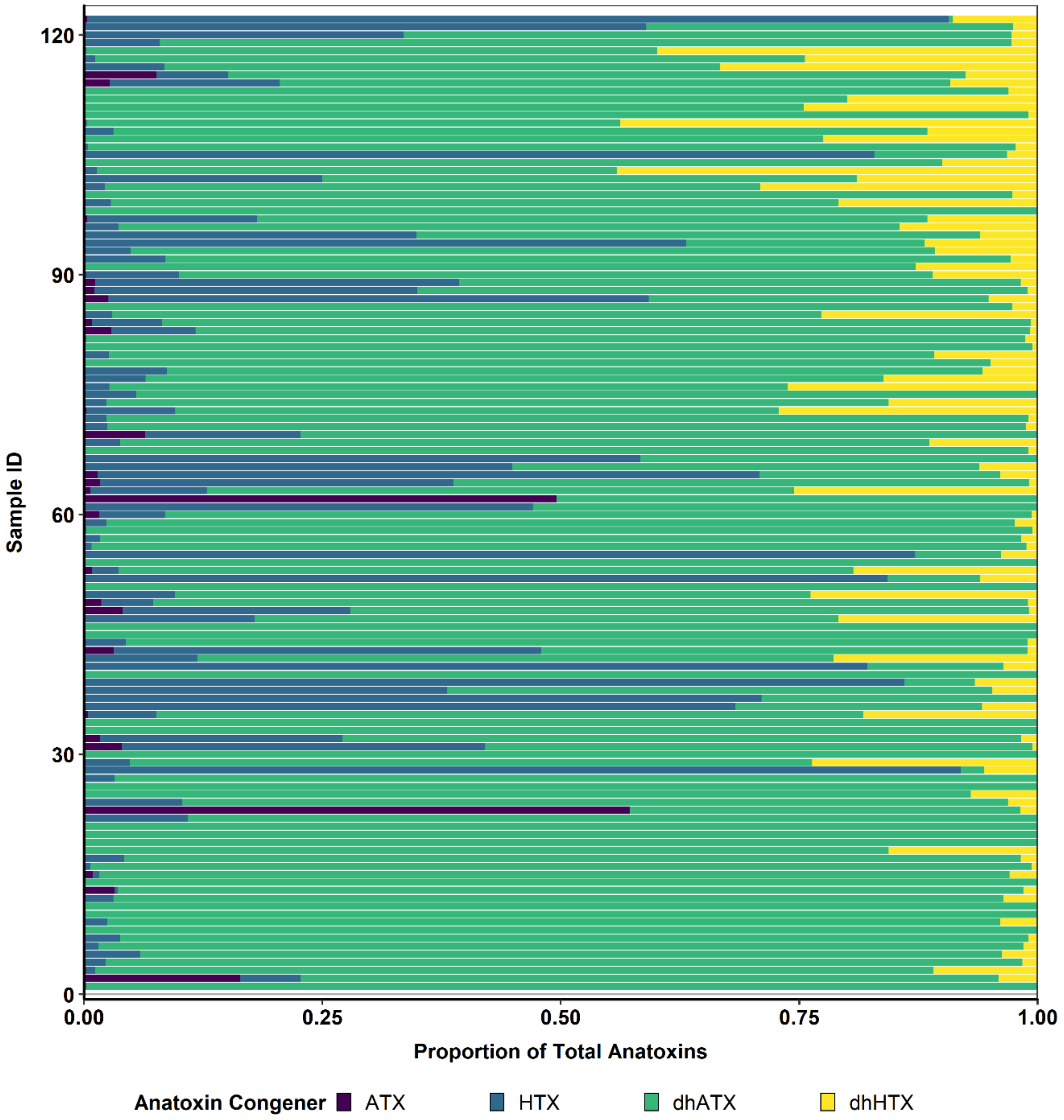

Figure S1. Proportions of each of the four anatoxin congeners anatoxin (ATX), homoanatoxin (HTX), dihydroanatoxin (dhATX) and dihydrohomoanatoxin (dhHTX) for each of the environmental samples. Sample ID are in order from the lowest anatoxin quota (sample ID 1) to highest anatoxin quota (sample ID 122).
